# Dual Inhibition of PARP and the Intra-S/G2 Cell Cycle Checkpoints Results in Highly Effective Radiosensitization of HPV-Positive HNSCC Cells

**DOI:** 10.3389/fonc.2021.683688

**Published:** 2021-07-20

**Authors:** Katharina Hintelmann, Thomas Berenz, Malte Kriegs, Sabrina Christiansen, Fruzsina Gatzemeier, Nina Struve, Cordula Petersen, Christian Betz, Kai Rothkamm, Agnes Oetting, Thorsten Rieckmann

**Affiliations:** ^1^ Department of Otorhinolaryngology, University Medical Center Hamburg-Eppendorf, Hamburg, Germany; ^2^ Department of Radiotherapy, University Medical Center Hamburg-Eppendorf, Hamburg, Germany; ^3^ Mildred-Scheel Cancer Career Center HATRICs4, University Medical Center Hamburg-Eppendorf, Hamburg, Germany

**Keywords:** head and neck cancer, human papillomavirus (HPV), molecular targeting, radiotherapy, radiosensitization, PARP, Wee1, Chk1

## Abstract

In head and neck squamous cell carcinoma (HNSCC), tumors positive for human papillomavirus (HPV) represent a distinct biological entity with favorable prognosis. An enhanced radiation sensitivity of these tumors is evident in the clinic and on the cellular level when comparing HPV-positive and HPV-negative HNSCC cell lines. We could show that the underlying mechanism is a defect in DNA double-strand break repair associated with a profound and sustained G2 arrest. This defect can be exploited by molecular targeting approaches additionally compromising the DNA damage response to further enhance their radiation sensitivity, which may offer new opportunities in the setting of future de-intensified regimes. Against this background, we tested combined targeting of PARP and the DNA damage-induced intra-S/G2 cell cycle checkpoints to achieve effective radiosensitization. Enhancing CDK1/2 activity through the Wee1 inhibitor adavosertib or a combination of Wee1 and Chk1 inhibition resulted in an abrogation of the radiation-induced G2 cell cycle arrest and induction of replication stress as assessed by *γ*H2AX and chromatin-bound RPA levels in S phase cells. Addition of the PARP inhibitor olaparib had little influence on these endpoints, irrespective of checkpoint inhibition. Combined PARP/Wee1 targeting did not result in an enhancement in the absolute number of residual, radiation induced 53BP1 foci as markers of DNA double-strand breaks but it induced a shift in foci numbers from S/G2 to G1 phase cells. Most importantly, while sole checkpoint or PARP inhibition induced moderate radiosensitization, their combination was clearly more effective, while exerting little effect in p53/G1 arrest proficient normal human fibroblasts, thus indicating tumor specificity. We conclude that the combined inhibition of PARP and the intra-S/G2 checkpoint is a highly effective approach for the radiosensitization of HPV-positive HNSCC cells and may represent a viable alternative for the current standard of concomitant cisplatin-based chemotherapy. *In vivo* studies to further evaluate the translational potential are highly warranted.

## Introduction

In locally advanced squamous cell carcinoma of the head and neck (HNSCC), positivity for human papillomavirus (HPV) confers a favorable prognosis, especially for patients with tumors located in the oropharynx (OPSCC) (1, 2). Standard treatment of locally advanced disease is cisplatin-based chemoradiation, either in the primary setting or as adjuvant treatment after surgery. The combination of high cure rates but often dramatic toxicity under these regimes has resulted in the development of various clinical trials testing de-intensification approaches, and some early phase trials have reported promising results (3–7). Two phase 3 trials, however, which together recruited more than 1,000 patients, concordantly reported inferiority of the rather cautious de-intensification concept of exchanging cisplatin for the also approved anti-EGFR antibody cetuximab under maintenance of the full radiation dose (8, 9). In line with these negative clinical results, we had previously shown that cetuximab completely fails to radiosensitize HPV-positive HNSCC cells *in vitro* (10). This clearly urges caution and speaks in favor of careful preclinical evaluation of novel agents and concepts.

A way to very directly induce radiosensitization is the molecular targeting of proteins involved in the DNA damage response (DDR) and DNA repair. Poly(ADP-ribose) polymerase 1 (PARP1) is responsible for poly(ADP-ribose) polymerization at the sites of DNA damage, which marks the lesion and recruits further DNA repair factors. PARP1 is involved in single-strand break repair but also in double-strand break (DSB) repair *via* the alternative end-joining (alt-EJ) backup DSB repair pathway (11, 12). Sole PARP inhibition is especially effective in tumors with a severe deficiency in homologous recombination (HR). Following the well-known concept of synthetic lethality, PARP inhibition increases the need for effective HR by interfering with the repair of intrinsic single-strand lesions and PARP-trapping at the break sites. Upon collision with replication forks, these structures can lead to the formation of one-ended DSBs, the repair of which requires HR (13, 14). Ionizing radiation induces both single- and double-strand breaks, and PARP-inhibitors are well known radiosensitizers (15).

Cell cycle checkpoints constitute another important factor in the response towards irradiation, providing more time for DNA repair before entering S-phase or mitosis in order to avoid mutations and especially mitotic cell death (16). In HNSCC, the majority of HPV-positive and -negative tumors are functionally deficient for p53 and subsequently also for the G1-S cell cycle checkpoint, increasing the dependence on the G2-M checkpoint. Reduction of the radiation-induced G2 arrest can be achieved by inhibition of the ATR/Chk1/Wee1 axis, as the inhibition of any of these kinases finally counteracts Wee1-mediated inhibitory phosphorylation of cyclin dependent kinase 1 (CDK1), which, in its active state will continue to drive G2-M transition (16, 17). Premature mitotic entry and induction of severe replication stress are further therapeutic effects resulting from enhanced CDK1 and CDK2 activity upon inhibition of the ATR/Chk1/Wee1 axis also without irradiation (18–20).

We and others have demonstrated that PARP inhibition as well as inhibition of radiation induced cell cycle checkpoints *via* targeting of Chk1, ATR, or Wee1 can radiosensitize HPV-positive HNSCC cells (10, 21–25). Different mechanisms may account for the observed sensitization. HPV-positive HNSCC cells are described to rely on PARP-dependent alt-EJ (26, 27) and to be defective in homologous recombination (HR) (27–31). Due to an ineffective DSB repair, these cells further rely on an especially profound and long lasting radiation-induced G2 arrest for the repair of radiation-induced DSBs before the critical passage through mitosis (21, 22, 32, 33). Apart from interfering with G2 arrest, the inhibition of Wee1, Chk1, or ATR can directly compromise the ability to perform HR (34–36) and the induction of replication stress, which is to a large extent caused by nucleotide shortage due to unrestrained CDK activity and enhanced origin firing (18), that may create an unfavorable environment for DNA repair in S phase. Given these potential S/G2 phase-based mechanisms, it is easily imaginable that the combined inhibition of PARP and the S/G2 cell cycle checkpoints could be an especially effective treatment option for HPV-positive HNSCC cells, and its radiosensitizing effect has already been demonstrated in preclinical studies in a number of other cancer entities (37, 38). Against this background, we tested the combined inhibition of PARP and the S/G2 cell cycle checkpoint in intrinsically DSB repair-compromised HPV-positive HNSCC cells using clinically relevant inhibitors, all of which are already being tested in combination with radiotherapy in clinical trials in HNSCC.

## Material and Methods

### Cells and Cell Culture

All cell lines were grown in RPMI (Sigma-Aldrich, St. Louis, MO, USA) supplemented with 10% fetal bovine serum (FBS) (Biochrom AG, Berlin, Germany) at 37°C, 5% CO_2_ and 100% humidification. HPV-positive HNSCC cells UD-SCC-2, UM-SCC-47 and UPCI-SCC-154, UPCI-SCC-90, 93VU-147T, UT-SCC-45, and normal human fibroblasts F184 were described previously (21, 33, 39). Tumor cell lines were identified by a short tandem repeat multiplex assay (Applied Biosystems, Waltham, MD, USA). PARP inhibition was performed using 1 µM olaparib (MyBiosource, San Diego, CA, USA). Wee1 inhibition was performed using 240 nM adavosertib (Selleckchem, Houston, TX, USA) and combined Wee1/Chk1 inhibition was performed at a dose of 60 nM adavosertib and 1 nM prexasertib (MedChemExpress, Monmouth Junction, NJ, USA) unless stated otherwise. Supplementation with nucleosides (EmbryoMax 100×, Sigma-Aldrich, St. Louis, MO, USA) was performed at a final dilution of 1/12.5.

### Cell Proliferation

For cell proliferation analysis, cells were seeded into T25 cell culture flasks and after 4 h treated with inhibitors. The numbers of resulting cells were assessed after 5 days using a Coulter counter (Beckmann-Coulter, Brea, CA, USA).

### Cell Cycle Assessment

Cells were harvested, fixed with 70% ethanol, briefly washed with PBS/0.2% Triton X-100, and subsequently incubated with PBS/1% BSA/0.2% Triton X-100/DAPI (4′,6-Diamidin-2-phenylindol, 1 µg/ml) for 30 min at room temperature in the dark. Cells were washed once with PBS/0.2% Triton X-100, and flow cytometric analysis was performed using a MACSQuant10 with MACSQuantify Software (Miltenyi Biotec, Bergisch Gladbach, Germany). The proportion of cells in the respective cell cycle phases was calculated using ModFit LT™ software (Verity Software House, Topsham, ME, USA).

### X-Irradiation

Cells were irradiated at room temperature with 200 kV X-rays (Gulmay RS225, Gulmay Medical Ltd., Suwanee, GA, USA; 200 kV, 15 mA, 0.8 mm Be + 0.5 mm Cu filtering; dose rate of 1.2 Gy/min).

### DSB Reporter Gene Assay

Exponentially growing HNSCC cells containing stably integrated copies of the previously described GFP-based HR or NHEJ reporter plasmids pGC or pEJ (40) were transfected with an I-SceI expression vector for targeted DSB induction using Fugene HD (Promega, Fitchburg, WI, USA). Six hours post transfection, the medium was exchanged and supplemented with inhibitors or solvent (DMSO) as indicated, followed by another exchange plus supplementation 24 h post transfection. At 48 h post transfection, the cells were harvested and assessed for GFP expression by flow cytometry using a FACS Canto with FACS Diva software (Becton Dickinson, Franklin Lakes, NJ, USA). The gating of GFP-positive cells was set according to the negative control (Fugene HD + empty vector). Rates of DSB repair (% GFP-positive cells) were normalized to the respective transfection efficiency of the individual experiment as determined by parallel transfection with a GFP-expression vector (pEGFP-N1).

### Immunofluorescence

Cells grown on glass cover slips were fixed with PBS/4% formaldehyde for 10 min, and permeabilized/blocked for 1 h or overnight with PBS/1% BSA/0.2%Triton X-100. The cells were subsequently incubated for 1 h at room temperature with the primary antibodies [mouse anti-53BP1 (clone BP13, Millipore, Billerica, MA, USA); rabbit anti-geminin (#10802-1-AP, Proteintech, Manchester, UK)] in blocking solution, washed four times with PBS/0.5% BSA/0.1% Triton X-100 before incubation with the secondary antibodies plus DAPI (1 µg/ml) and were then washed again four times before mounting with Vectashield mounting medium (Vector Laboratories, Burlingame, CA, USA). Cells were inspected using an AxioObserver Z1 fluorescence microscope with ApoTome and Axiovision Software (Zeiss, Oberkochen, Germany). 53BP1 foci per nucleus were manually counted using stack images in maximum intensity projection. Nuclei with ≥20 foci were scored as “20”.

### Flow Cytometric Protein Quantification

Flow cytrometric measurement of relative protein staining intensity per cell in relation to the cell cycle phase was performed on either a FACS Canto with FACS Diva Software (Becton Dickinson, Franklin Lakes, NJ, USA) using FxCycle FarRed (Molecular Probes, Eugene, OR, USA) as nuclear counterstain or on a MACSQuant10 with MACSQuantify and Flowlogic Software (Miltenyi Biotec, Bergisch Gladbach, Germany & Inivai, Mentone Victoria, Australia) using DAPI as nuclear counter stain. In brief, cells were harvested, fixed with PBS/4% formaldehyde for 10 min, and then permeabilized and blocked with PBS/1% BSA/0.2% Triton X-100 for a minimum of 1 h. The cells were subsequently incubated (1 h; room temperature) with the primary antibody [rabbit anti-P-Histone3 (#06-570, Millipore, Billerica, MA, USA), mouse-anti-*γ* H2AX antibody (clone JBW301, Millipore, Billerica, MA, USA), and mouse anti-RPA32 (clone ME34, Santa Cruz, Santa Cruz, CA, USA)] in blocking solution, washed three times with PBS/0.5% BSA/0.1% Triton X-100 before incubation (1 h; room temperature) with the second antibody and were then washed again three times. DNA counterstaining was either performed with DAPI added to the secondary antibody or with FxCycle FarRed (Molecular Probes, Eugene, OR, USA) plus 300 ng/ml RNAse A and 0.2% Triton X-100 for 30 min at room temperature in the dark following the last washing step.

In case of RPA staining, the cells were pre-extracted after trypsinizaton by gentle resuspension (wide bore tips) of the harvested cell pellet in 500 µl ice cold PBS/0.1% Triton X-100/1 mM DTT followed by gentle shaking in horizontally placed reaction tubes on ice for 10 min. Afterwards, 1 ml cold PBS/1% BSA/1mM DTT was added, tubes were inverted several times, and the pre-extracted cells were collected in a pre-cooled centrifuge (5 min, 400 g). After discarding the supernatant, the pre-extracted cells were resuspended (wide bore tips) in PBS/4% formaldehyde and fixed for 10 min at room temperature before regular subsequent staining procedures as described above.

### Colony Formation Assay

Radiosensitization was determined using delayed plating colony formation assay. Exponentially growing cells were treated with inhibitor and irradiated after 2 h of incubation. Twenty-four hours post irradiation the cells were seeded in defined numbers into T25 cell culture flasks without addition of inhibitors. Incubation time until colony formation varied between cell lines from 2 to 4 weeks; irradiated samples of HPV-positive cell lines were allowed to grow for an extended period of time, as colony formation was apparently delayed. The number of colonies containing more than 50 cells was assessed. In the case of UM-SCC-47, the cell number was adjusted to 5000 by addition of feeder cells (UM-SCC-47; 20 Gy) to support plating efficiency, and for UPCI-SCC-154 and F184 the medium was changed to a 1:1 mixture of RPMI/10% FBS and Amniomax C-100 medium/7.5% Amniomax Supplement (both Gibco, Thermo Fisher Scientific, Waltham, MA, USA)/7.5% FBS one (F184) or three (UPCI-SCC-154) weeks after seeding to facilitate colony formation.

### Data Evaluation

Data analyses were performed using Excel (Microsoft, Redmond, WA, USA) and GraphPad Prism 6 (GraphPadSoftware, San Diego, CA, USA). All experiments were performed at least three times, and single experiments always contained the full set of substances and radiation doses as indicated. Values presented are mean ± SD unless indicated otherwise. Two-tailed Student’s t-test was used to assess statistically significant differences using GraphPad Prism 6.

## Results

**Figure 1:**
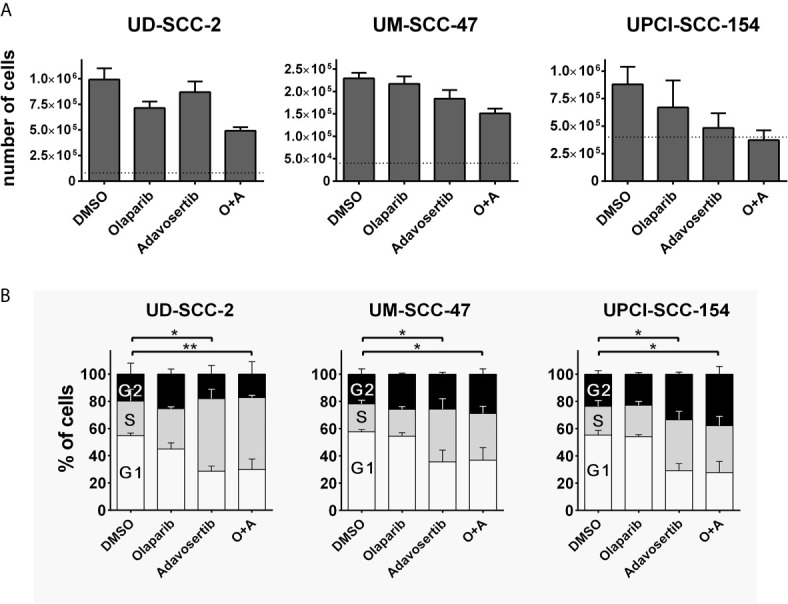
Interactions of PARP and Wee1 inhibition. **(A)** Proliferation. Cells were seeded and after 4 h treatment with inhibitors as indicated. Five days later the respective numbers of cells were assessed. Dotted lines indicate the number of cells seeded. Adavosertib: UD-SCC-2 & UM-SCC-47, 120 nM; UPCI-SCC-154, 60 nM. Olaparib: all strains 500 nM. **(B)** Cell cycle. Cells were seeded and on the next day treated with the respective inhibitors. After 24 h, the cells were fixed and subjected to DAPI staining and flow cytometric assessment of cell cycle distribution. Adavosertib: UD-SCC-2 & UPCI-SCC-154, 480 nM; UM-SCC-47, 960 nM. Olaparib: all strains 1 µM. Statistical evaluation was performed for changes in the S-phase population; addition of olaparib did not induce any significant changes. Asterisks depict significant differences with * and ** indicating p < 0.05 and p < 0.01, respectively (two-tailed Student’s t-test).

To assess whether the dual inhibition of PARP and Wee1 may exert some additive or synergistic effects in HPV-positive HNSCC cells, we tested a combination of the PARP inhibitor olaparib and the Wee1 inhibitor adavosertib (MK-1775/AZD-1775) with regard to cell proliferation and cell cycle distribution. To this end we used individual inhibitor doses that previously demonstrated moderate effects on their own with regard to the respective cell lines and endpoints or a maximum concentration of 1 µM olaparib in the cell cycle analyses, which was previously proven sufficient to completely suppress the poly(ADP)-ribosylation of HPV-positive HNSCC cells upon H_2_O_2_ treatment (10, 22). Regarding proliferation we observed several statistically significant differences and the generally strongest reduction under combined inhibition but without a clear hint for a meaningful synergistic effect (**Figure 1A**). Regarding cell cycle distribution, adavosertib induced an accumulation of cells in the S-phase, indicative of replication stress, while olaparib had little effect on its own or when added to Wee1 inhibition (**Figure 1B**).

### Radiation-Induced Cell Cycle Arrest

**Figure 2:**
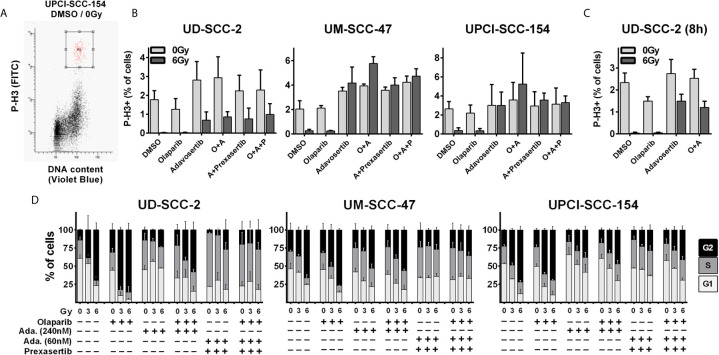
Radiation-induced G2 arrest. **(A–C)** Fraction of mitotic cells. Exponentially growing cells were treated for 2 h with the inhibitors as indicated (olaparib: 1 µM; adavosertib: 240 nM; adavosertib + prexasertib: 60 nm + 1 nM, respectively), before irradiation with 0 or 6 Gy. Five or eight hours after irradiation cells were fixed and stained for phospho-histone H3 (P-H3+) to assess the number of mitotic cells. **(A)** Gating. **(B)** Quantification of the mitotic fraction at 5 h after irradiation. **(C)** Quantification at 8 h after irradiation. **(D)** Long term G2 arrest. Cells were treated and irradiated as in **(A–C)**. Twenty-four hours after irradiation the cells were fixed, and the cell cycle distribution was assessed by DAPI staining and flow cytometry.

While the previous results did not indicate prominent synergistic effects, we further tested dual PARP and S/G2 checkpoint inhibition combined with ionizing irradiation. To assess a direct effect on the radiation-induced G2 arrest, we quantified the amount of phospho-histone H3 positive mitotic cells 5 h after 6 Gy ± inhibitor treatment (**Figure 2A**). Sole adavosertib treatment (240 nM) increased the rate of mitotic cells in two cell lines, indicating unscheduled mitotic entry upon Wee1 inhibition as previously described (41). Irradiation largely blocked mitotic entry in all strains irrespective of olaparib treatment (1 µM). Adavosertib completely suppressed this G2 arrest, except for UD-SCC-2 cells, where it could only partially override checkpoint execution (**Figure 2B**). Additionally testing a later time point of 8 h post irradiation, adavosertib treatment ± olaparib further relieved UD-SCC-2 cells from the radiation-induced G2 checkpoint (**Figure 2C**). We had previously shown that Wee1 inhibition activates Chk1, which could in part compensate the reduction in Wee1 activity and, indeed, dual inhibition was effective at profoundly reduced doses (22). As low dose dual Wee1/Chk1 inhibition may potentially offer a clinical alternative to high dose single inhibitor treatment, we also included a combination using especially low concentrations of 60 nM adavosertib and 1 nM of the Chk1/2 inhibitor prexasertib, which showed limited effectiveness on their own (**Supplementary Figure S1**). This dual checkpoint inhibition resulted in checkpoint abrogation comparable to the higher dose (240 nm) of sole adavosertib treatment irrespective of the addition of olaparib in all strains (**Figure 2B**).

As HPV-positive HNSCC cells show prolonged G2-checkpoint responses due to an inefficient DNA DSB repair (33), we further assessed cell cycle distribution at a later time point of 24 h after irradiation where all cell lines demonstrated profound radiation-induced G2 arrest (**Figure 2D**). In line with the short term experiments described above, adavosertib treatment reduced the amount of radiation-induced G2 arrest also at 24 h after irradiation but not to the full extent. The combination of adavosertib and prexasertib also reduced G2 arrest and partly increased the amount of S phase cells, suggesting severe replication stress. Addition of olaparib to adavosertib ± prexasertib did not induce any further accumulation in S-phase irrespective of radiation. In UD-SCC-2 cells, sole olaparib treatment resulted in a clear increase of cells in G2, especially after irradiation but also at baseline. In UM-SCC-47 and UPCI-SCC-154 the increase was subtle but highly reproducible, which is in line with enhanced DNA damage levels after PARP inhibition as frequently reported (**Supplementary Figure S2A**) (42–44). Enhanced damage levels are further supported by higher intensity of the DNA damage marker *γ*H2AX in cells residing in radiation-induced G2-arrest after olaparib treatment in all three cell lines (**Supplementary Figure S2B**).

For all the following experiments, we continued with concentrations of 1 µM olaparib and 240 nM adavosertib or, alternatively, the reduced concentration of 60 nM adavosertib combined with 1 nM prexasertib, which demonstrated similar G2 checkpoint abrogation in these assays.

### Replication Stress

Unscheduled activation of dormant origins and subsequent nucleotide depletion is described as a mechanism of antitumor activity through Wee1 and/or Chk1 inhibition (18, 20). This leads to replication stress and, if severe, S-phase arrest as partially observed for the combined Wee1/Chk1 inhibition described above. Chk1 is further described as a replication fork protection factor (45) and PARP1, apart from its functions in DNA repair, was reported to be involved in the restart of stalled replication forks and Chk1-dependent S-phase checkpoint activation and fork protection (46–49).

**Figure 3:**
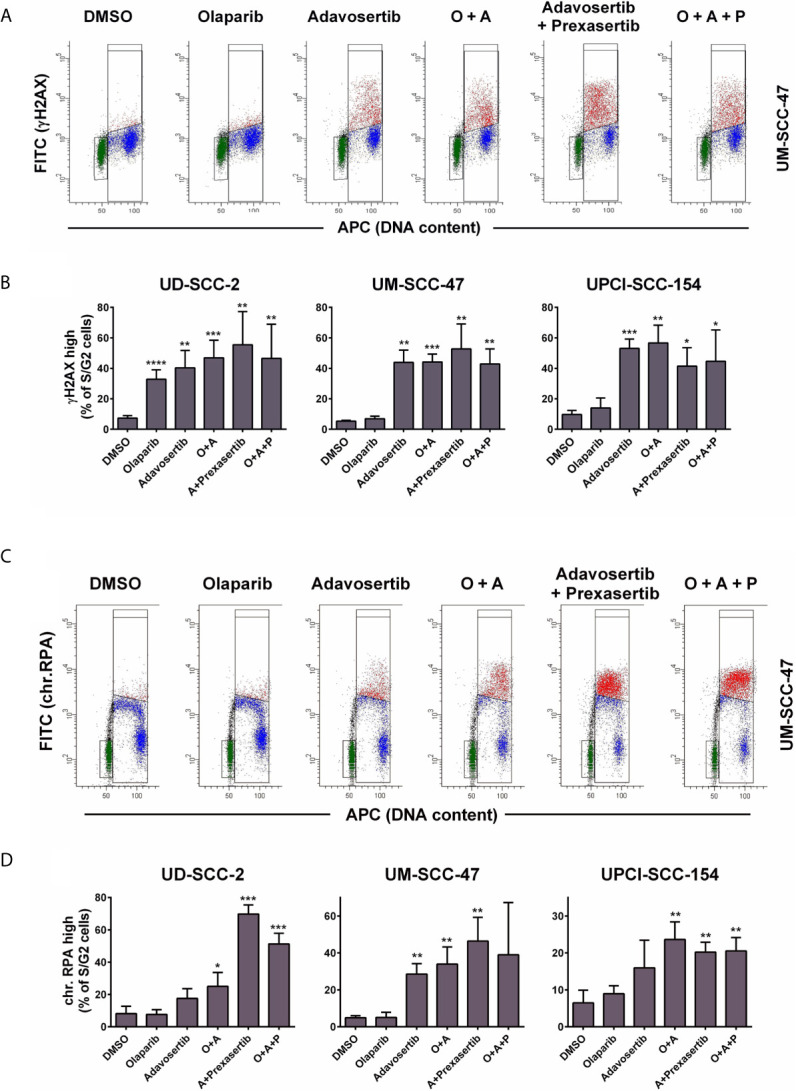
Effect of PARP and intra-S/G2 checkpoint inhibition on γH2AX and chromatin-bound RPA staining intensity. Cells were treated with inhibitors as indicated for 24 h before fixation, staining, and flow cytometric measurements. In case of RPA staining the cells were pre-extracted before fixation. **(A)** Examples of the flow cytometric measurement of *γ*H2AX. Gates are set to select cells in G1 (green), in S/G2 (red & blue) or cells in S/G2 with enhanced **γ**H2AX levels (red). **(B)** Fraction of S/G2 phase cells that demonstrate enhanced **γ**H2AX levels. **(C)** Examples of the flow cytometric measurement of chromatin-bound RPA, which is highest in the replicative S-phase. Gates are set to select cells in G1 (green), in S/G2 (red and blue) or cells in S with enhanced RPA levels (red). **(D)** Fraction of S/G2 phase cells that demonstrate enhanced RPA staining levels. Asterisks depict significant differences to solvent (DMSO) treatment with *, **, *** and **** indicating p < 0.05, p < 0.01, p < 0.001, and p < 0.0001, respectively (two-tailed Student’s t-test).

In S-phase, cell stretches of single-strand DNA (ssDNA) upon replication fork stalling as well as DSBs upon replication fork collapse are recognized through the related ATR and ATM kinases, and such areas are subsequently decorated by **γ**H2AX. In line with these mechanisms, the inhibition of Wee1 as well as the combined inhibition of Wee1/Chk1 resulted in a strong increase in *γ*H2AX signal intensity in S and partly G2 phase cells. However, neither olaparib alone nor the addition of olaparib to Wee1 or to Wee1/Chk1 inhibition resulted in any substantial increase in **γ**H2AX levels with the exception of sole addition in UD-SCC-2 cells. Here, a considerable number of cells demonstrated higher *γ*H2AX levels, but the rise in signal intensity was very modest (**Figures 3A, B** and **Supplementary Figure S3A**). Although less uniform than the **γ**H2AX staining, the results were in principle confirmed when assessing the amounts of chromatin-bound RPA, which, as the primary ssDNA binding and protection factor, represents a very direct and robust marker for replication stress (50) (**Figures 3C, D**). Notably here, in UD-SCC-2 and UPCI-SCC-154, sole Wee1 inhibition resulted in a more moderate induction of RPA signal intensity compared to combined Wee1/Chk1 inhibition, in line with the stronger accumulation in the S-phase described above (**Supplementary Figure S3B**). Adding the PARP inhibitor did not prominently change the amount of cells positive for **γ**H2AX or chromatin-bound RPA.

Together these results demonstrate that under Wee1 and especially Wee1/Chk1 inhibition S phase cells will have to repair radiation induced DNA damage under conditions of replication stress and with a severely reduced ability to halt the cell cycle in G2 and therefore without extra time for DNA repair before the critical passage through mitosis. Additional inhibition of PARP did not prominently impact on replication stress or inhibition of G2 arrest according to the endpoints measured.

### DSB Repair

The reduced DNA DSB repair capacity of HPV-positive HNSCC cells has been frequently ascribed to a defect in the DNA repair pathway homologous recombination (HR) (28–31) and also a switch towards the error prone alt-EJ pathway has been reported (26, 27). As PARP1 is a key component of the latter (12) and Wee1 has been described as a relevant HR factor (35), we tested the influence of PARP- and Wee1 inhibition on NHEJ and HR using established GFP-based reporter gene constructs stably integrated in HPV-positive UD-SCC-2 and UPCI-SCC-154 cells (51) (**Supplementary Figures S4A, B**). Although the pEJ construct can interrogate classical NHEJ and alt-EJ repair (52), PARP inhibition did not reduce the rate of measurable NHEJ in either cell line (**Supplementary Figure S4C**). Despite the reported HR defect of HPV-positive HNSCC cells, we had also been able to establish UD-SCC-2 and UPCI-SCC-154 HR reporter cells. Unexpectedly, Wee1 inhibition did not reduce the rate of HR repair as assessed through the pGC reporter construct and the combination with PARP inhibition even increased the rate of GFP-positive cells (**Supplementary Figure S4D**).

**Figure 4:**
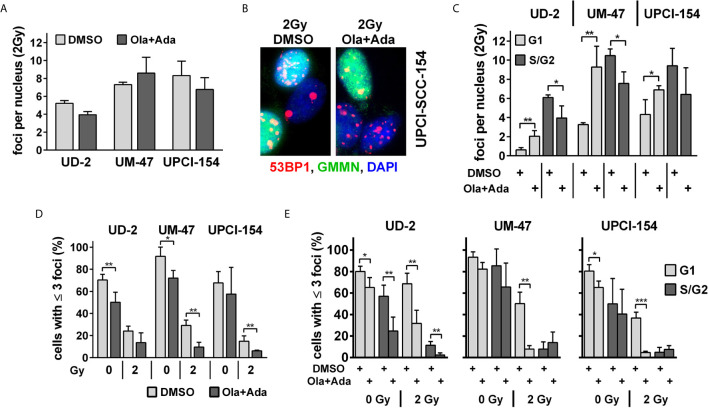
Effect of PARP and Wee1 targeting on DSB repair. **(A)** Quantification of radiation-induced nuclear 53BP1 foci at 24 h after 2 Gy irradiation. Counts were normalized to the DNA content of the respective cell lines as assessed previously (33), foci numbers in non-irradiated controls were subtracted. **(B)** Example of immunofluorescence co-staining of 53BP1 and the S/G2 phase marker geminin (GMMN). **(C)** Quantification of radiation-induced nuclear 53BP1 foci with respect to the cell cycle phase as determined by geminin co-staining. Foci numbers in non-irradiated controls were subtracted. **(D)** Fraction of cells with ≤3 53BP1 nuclear foci. **(E)** Fraction of cells with ≤3 53BP1 nuclear foci with respect to cell cycle phase as determined by geminin co-staining. Significant changes are indicated with *, ** and *** indicating p < 0.05, p < 0.01, and p < 0.001, respectively (two-tailed Student’s t-test).

In line with the reporter gene assay results, we also did not observe an enhancement of residual 53BP1 nuclear foci as markers of unrepaired DSBs at 24 h after irradiation with 2 Gy under combined PARP/Wee1 inhibition in UD-SCC-2 and UPCI-SCC-154 and only a slight, non-significant increase in UM-SCC-47 (**Figure 4A** and **Supplementary Figure S5A**). We did, however, observe a common phenotype regarding the distribution of foci with respect to the cell cycle phase as determined by geminin co-staining, which marks cells in S and G2 phases (**Figure 4B**). In all cell lines, the average foci number in G1 increased significantly upon combined PARP/Wee1 inhibition, whereas foci in S/G2 phase cells decreased (**Figure 4C** and **Supplementary Figure S5B**). In line with the respective cell cycle data (**Figure 2**), this underscores that under combined inhibition cells with unrepaired DSBs exit G2 arrest and take the critical passage through mitosis despite the enhanced risk of acute and delayed mitotic cell death. In general, cells with low numbers of residual radiation-induced DSBs are the ones most likely to survive and the fraction of such potentially surviving cells after 2 Gy was decreased in all strains upon dual PARP/Wee1 inhibition, albeit in UD-SCC-2 slightly missing significance (p = 0.0777) (**Figure 4D**). Regarding cell cycle, this reduction was observed in the G1-phase in all strains, again underpinning premature mitotic passage (**Figure 4E**). Surprisingly, in UD-SCC-2 the fraction of cells with few residual foci was also significantly reduced in S/G2 phase cells upon dual inhibition, despite the overall decrease in average foci numbers in this fraction (**Figure 4C**).

### Radiosensitization

**Figure 5:**
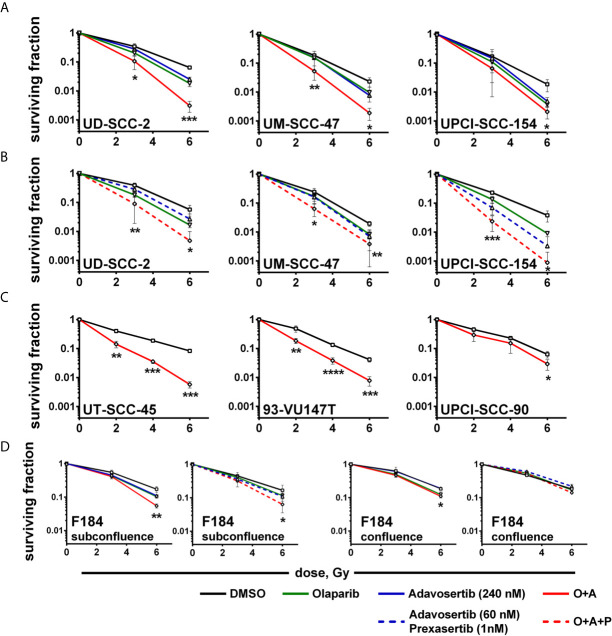
Radiosensitization. Exponentially growing cells were seeded and on the next day treated with inhibitors as indicated and irradiated 2 h thereafter; 24 h later, irradiated cells were seeded in low, defined numbers for colony formation. **(A)** Radiosensitization of HPV-positive HNSCC cells using dual PARP/Wee1 inhibition or **(B)** combined PARP/Wee1/Chk1 inhibition. **(C)** Validation of radiosensitization through combined PARP/Wee1 inhibition using three additional HPV-positive HNSCC cell lines. **(D)** Effect on normal human fibroblasts as an example of normal tissue cells. Significance was assessed for solvent control *vs*. combined PARP + S/G2 checkpoint inhibition. In case of a statistically significant difference the respective dose points are marked with asterisks with *, **, *** and **** indicating p ≤ 0.05, p ≤ 0.01, p ≤ 0.001, and p ≤ 0.0001, respectively (two-tailed Student’s t-test).

So far while we did not observe clear hints pointing towards enhanced cytotoxicity when adding a PARP inhibitor to intra-S/G2 checkpoint inhibition, radiosensitization through PARP inhibition is clearly established owing to an enhanced induction of replication-induced one-ended DSBs, the inhibition of alt-EJ and further mechanisms (53). Moreover, we had previously observed highly effective radiosensitization in HPV-positive HNSCC cells when combining olaparib with the Chk1 inhibitor PF-00477736 (10). In line with these results, a significant reduction of colonies indicating radiosensitization was now observed upon combined PARP/Wee1 inhibition as compared to single inhibitor usage (**Figure 5A**). Highly similar results were obtained when replacing the 240 nM adavosertib treatment with 60 nM adavosertib/1 nM prexasertib (**Figure 5B**). To further estimate whether radiosensitization occurs in a majority of HPV-positive HNSCC cells, we tested dual PARP/Wee1 targeting in three additional strains, all of which were also sensitized, two very effectively and UPCI-SCC-90 to less extent (**Figure 5C**). To assess tumor specificity, we further tested dual targeting in p53/G1 arrest proficient normal human fibroblasts. In a proliferative state, fibroblasts were radiosensitized by combined inhibition but to a lesser extent than five of the six HPV-positive tumor cell lines. In confluent cultures, the effect of intra-S/G2 checkpoint targeting was completely lost, and radiosensitization was marginal or absent (**Figure 5D** and **Supplementary Table S1**). A comparison of the plating efficiency rates of the non-irradiated controls did not reveal a clear differential effect of the dual *vs.* the triple inhibition approach in HPV-positive HNSCC cells and virtually no reduction of survival in the normal fibroblasts (**Supplementary Figure S6**).

### Nucleoside Supplementation Counteracts Radiosensitization Through Wee1 but Not PARP/Wee1 Inhibition

**Figure 6:**
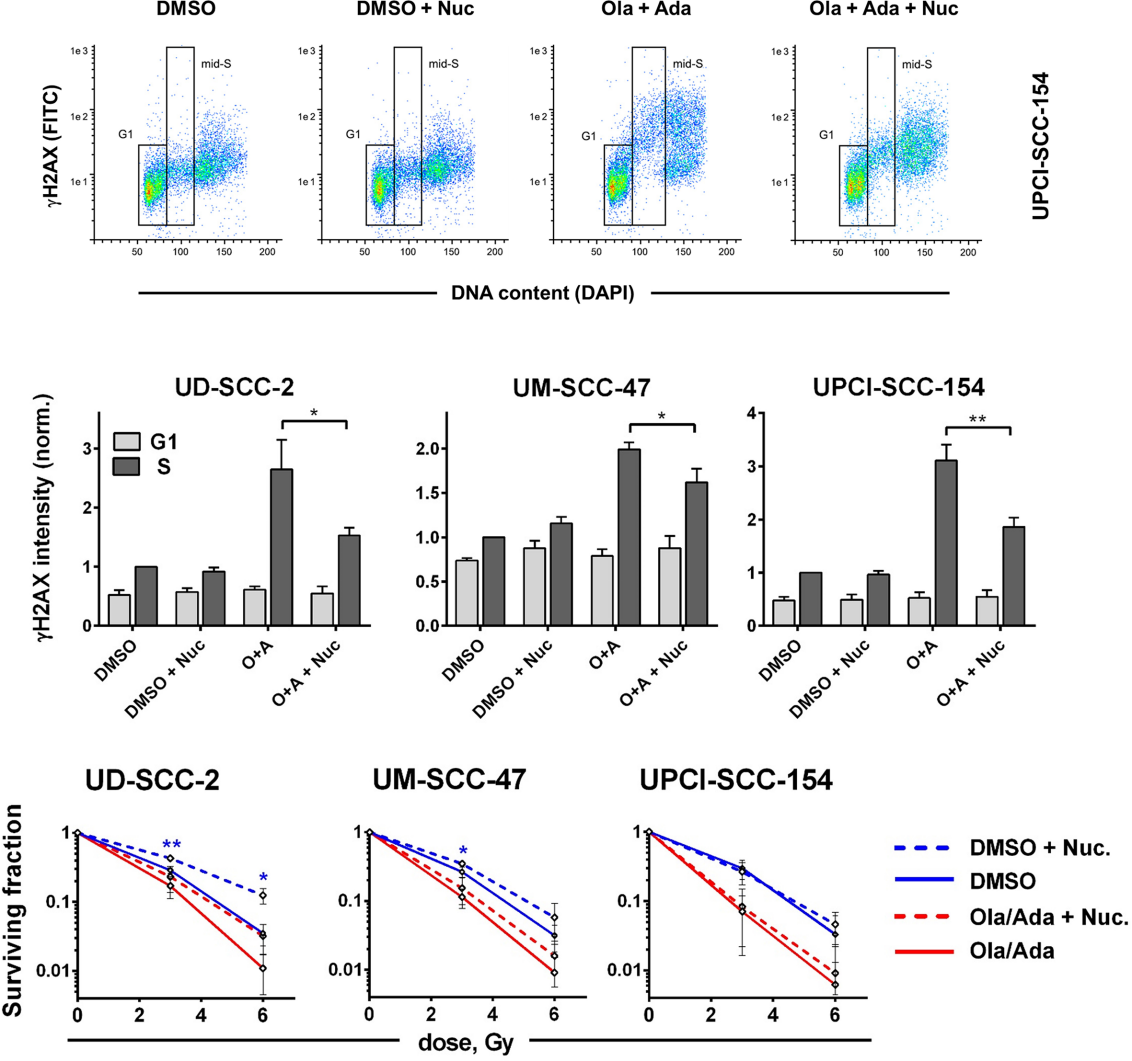
Cell line-dependent induction of radioresistance through nucleoside supplementation. Exponentially growing cells were treated with or without the combination of olaparib and adavosertib and with or without external nucleosides as indicated. **(A)** Example of gating for *γ*H2AX intensity measurement in G1 and mid-S phase cells as assessed by DAPI co-staining. The cells were fixed after 4 h of treatment and analyzed for *γ*H2AX induction by flow cytometry. **(B)** Bars depict the average median *γ*H2AX staining intensity of cells in G1 and mid-S phase. Values were normalized to the intensity of DMSO-treated mid-S phase cells of the respective experiments. Asterisks mark statistically significant differences upon nucleoside supplementation. **(C)** Two hours after addition of inhibitors ± nucleosides the cells were irradiated and after further 24 h seeded for colony formation without addition of inhibitors. Asterisks mark statistically significant differences in survival upon nucleoside supplementation, color indicates solvent controls or inhibitor treatment. Differences between DMSO treatment and dual inhibition without nucleoside supplementation (solid lines) were significant for all cell lines (not indicated). Significant changes are indicated with * and **indicating p ≤ 0.05 and p ≤ 0.01, respectively (two-tailed Student’s t-test).

We finally wanted to estimate to what extent the induction of replication stress may contribute to the profound radiosensitization upon combined treatment. As a shortage in nucleotides contributes to replication stress upon intra-S/G2 checkpoint inhibition, it can partly be compensated by external addition of nucleosides (18, 54). To test the effect in our cells, we analyzed *γ*H2AX levels in S-phase cells at 4 h after combined PARP/Wee1 inhibition, a time point corresponding to 2 h post irradiation in the colony formation assays when DSB repair would be highly active. We found *γ*H2AX levels to be induced by combined inhibition in S phase cells and partly suppressed by nucleoside supplementation. A substantial degree of induction and normalization was observed in UD-SCC-2 and UPCI-SCC-154 cells (**Figures 6A, B**). Despite these similarities, nucleoside supplementation did not influence radiation sensitivity in UPCI-SCC-154 but in UD-SCC-2 induced a quite clear trend towards radioresistance in the PARP/Wee1-inhibited samples (6 Gy: p = 0.0862). Unexpectedly, resistance was induced in the solvent-treated controls to a very similar extent reaching significance for the 6 Gy dose point (**Figure 6C**). In comparison, sole Wee1 inhibition induced a similar increase in *γ*H2AX levels, and nucleoside supplementation resulted in a pertinent normalization. In contrast to the situation under combined targeting, nucleoside supplementation counteracted adavosertib-mediated radiosensitization in UM-SCC-47 and UPCI-SCC-154, with no or little effect in the respective solvent-treated controls. Solely in UD-SCC-2, nucleoside supplementation exerted a similar effect on adavosertib and control treated cells (**Supplementary Figure S7**). So while these data strongly suggest that replication stress caused by nucleotide shortage can play a prominent role in the radiosensitization under sole Wee1 inhibition, they question a meaningful role for the radiosensitization under combined PARP/Wee1 inhibition in our cells.

The cause for radioresistance under nucleoside supplementation in solvent treated UD-SCC-2 cells currently remains elusive. In a set of pilot experiments, nucleosides increased the fraction of G1 at the cost of S phase cells in UD-SCC-2 and reduced their proliferation speed (**Supplementary Figures S8A, B**). Also especially in UD-SCC-2, the radiation-induced G2 arrest was diminished upon nucleoside supplementation, suggesting that fewer residual DSBs were present to trigger the G2 cell cycle checkpoint (**Supplementary Figure S8A**). Finally, analyses of residual DSBs under nucleoside supplementation *via* 53BP1 nuclear foci in UD-SCC-2 cells demonstrated an increase in the fraction of cells with few (≤3) foci after irradiation, in line with radioresistance induction. The effect was present and significant in both cells that were or were not actively replicating at the time of irradiation (**Supplementary Figure S8C**). Further analyses will be necessary to clarify this intriguing finding of radioresistance through nucleoside supplementation in otherwise unperturbed cells.

## Discussion

Inhibition of Wee1 by adavosertib was recently described as a highly effective single-agent treatment for HPV-positive HNSCC dependent on FOXM1 activation (55) and single agent radio- and chemosensitization through PARP, as well as through intra-S/G2 checkpoint inhibition, which was repeatedly demonstrated in HPV-positive HNSCC models (10, 21–23, 25, 30, 31, 56, 57). In this study we demonstrate a highly effective radiosensitization of HPV-positive HNSCC cells using dual inhibition of PARP and the S/G2 cell cycle checkpoint in five and moderate radiosensitization in one out of six cell lines tested. A similar result has recently been independently described for the HPV-positive strain UPCI-SCC-154 (24). Here it was suggested that the combination of PARP plus Chk1 inhibition is more effective in HPV-positive HNSCC cells, whereas the combination of PARP plus Wee1 inhibition is more effective in HPV-negative ones but the estimation was based on only one cell line per group. For this particular HPV-positive strain, we have indeed also observed an exceptionally strong radiosensitization when including a Chk1 inhibitor (**Figure 5**). The data are also in line with previous findings of strong radiosensitization using sole Chk1 and combined Chk1/Wee1 inhibition, but again the effect was only specific for UPCI-SCC-154 rather than for HPV-positive cells in general (22). Of note, this strain was also an outlier in the response to the particular Chk1 inhibitor PF-004776, but here demonstrated non-responsiveness for various endpoints, which further suggests irregularities (21). Effective radiosensitization through combined inhibition of PARP and the intra-S/G2 cell cycle checkpoint has also been described for other entities and for different approaches of checkpoint targeting, such as Chk1 or ATR inhibition (10, 58–60). The combination of PARP/Wee1 inhibition was previously tested in lung and pancreatic cancer cells with similarities but also some differences to our findings in HPV-positive HNSCC cells (61, 62). Contrasting these studies we did not observe inhibition of HR upon Wee1 inhibition in plasmid reconstruction assays and we neither observed a reduction of NHEJ upon PARP inhibition despite the reported enhanced usage of alt-EJ in HPV-positive HNSCC (26, 27). Furthermore, while replication stress was clearly evident upon intra-S/G2 checkpoint inhibition, we could not confirm an important role for the radiosensitization under combined inhibition, since for example in UD-SCC-2 targeting the intra-S/G2 checkpoint by combined Wee1/Chk1 inhibition induced replication stress more effectively than sole Wee1 inhibition but radiosensitization was highly similar (**Figures 2**, **3**, **5**). And while external nucleoside supplementation succeeded in partly relieving replication stress, it either failed to reduce radiosensitization (UPCI-SCC-154) or induced radioresistance in the solvent-treated controls to a similar extent as under combined PARP/Wee1 inhibition (UD-SCC-2) (**Figure 6**). In contrast, nucleoside supplementation demonstrated a pertinent reduction in replication stress and effectively counteracted radiosensitization upon sole Wee1 inhibition in two out of three cell lines tested (**Supplementary Figure S7**), suggesting additional mechanisms and a more robust radiosensitization upon combined inhibition. These findings are actually in line with previous reports, where the addition of nucleosides also counteracted radiosensitization under sole Wee1 (62, 63) but not under combined Wee1/PARP inhibition (62). Interestingly, nucleoside supplementation had also induced radioresistance in solvent treated samples in one of three (hepatocellular) carcinoma cell lines tested, while in NSCLC cells no results for the solvent treated controls were presented (62, 63). While clearly not the focus of this manuscript, our observation of profoundly enhanced radioresistance upon nucleoside supplementation in solvent-treated UD-SCC-2 cells is interesting and warrants future mechanistic investigations.

A puzzling finding of our study is the slight reduction in the overall number of 53BP1 foci upon combined treatment (**Figure 4**). In general, an enhancement in DNA damage in S/G2 phase upon PARP inhibition is very well established (13, 14, 64) and, accordingly, we observed an increase in G2 arrested cells and enhanced *γ*H2AX levels in G2 phase cells upon PARP inhibition and moderate radiosensitization here and previously (**Figures 2**, **5** and **Supplementary Figure S2**) (10). A possible explanation, in line with the cell cycle data and the shift in foci number from G2 to G1 phase cells (**Figures 2**, **4**) may be that overriding the otherwise long lasting G2 checkpoint can result in immediate mitotic catastrophe and cell elimination, preferentially of those cells with high damage and foci levels that would otherwise reside long enough in G2 to be scored. In line with this theory, the proportion of irradiated G2 phase cells with ≥20 53BP1 foci decreased in UD-SCC-2 and UPCI-SCC-154 upon combined inhibition (data not shown). Importantly, the fraction of cells with very low foci numbers was reduced upon dual inhibition in all cell lines tested. Overall, our results point towards a mechanism for radiosensitization driven by the abrogation of the, in HPV-positive HNSCC cells extensive, G2 cell cycle arrest in combination with the induction of additional DNA damage in S/G2 through PARP inhibition. While differences may exist in detail, the described effectiveness in different entities and by application of various checkpoint inhibitors clearly point towards a very robust radiosensitization of proliferating tumor cells by this combinatorial approach (37, 38). In contrast, normal fibroblasts, representing p53-proficient normal tissue cells, were only modestly affected in our study (**Figure 5D**), which indicates a fair degree of tumor specificity, especially given that many normal tissues do not or only slowly proliferate.

From the translational view, HPV-positive HNSCC may represent an especially promising entity for radiosensitization through molecular targeting. Patients possess a favorable prognosis and therefore targeting agents may not be added to concomitant chemotherapy (CT) but could rather replace CT and this should reduce, instead of increase, the risk of severe systemic side effects. Safe de-intensification of treatment is already the common goal in clinical trials for HPV-positive HNSCC. A major drawback, however, was the reported inferiority of cetuximab compared to cisplatin despite maintaining full dose radiotherapy in two phase 3 trials (65). These studies clearly highlight the need for effectiveness and thorough preclinical evaluation of molecular targeting approaches despite the overall favorable prognosis. In the frame of recent clinical data on de-intensification, promising initial results were obtained for reducing radiation dose in definitive chemoradiation and after induction chemotherapy (ICT) (3, 5, 7). In the frame of the latter, effective targeting may also be an alternative to adjuvant chemotherapy after ICT in the frame of risk-adapted, de-intensified radiotherapy and may evade potential chemoresistance mechanisms selected for or acquired during ICT. All inhibitors used in this study are already being tested in clinical trials in combination with radiotherapy in HNSCC (66, 67) (NCT02555644, NCT01758731, NCT02308072, NCT02585973). Olaparib is clinically approved in other entities, and the combination of adavosertib and radiotherapy (plus gemcitabine) was recently reported to yield promising results in pancreatic cancer (68). Moreover, combined treatment with olaparib and adavosertib as well as with prexasertib is also being clinically tested in a number of entities (NCT02576444, NCT02511795, NCT03579316, NCT03330847), albeit so far not in combination with radiotherapy. From our point of view, the clinical stage of the inhibitors available and the preclinical evidence provided in this study clearly warrant subsequent *in vivo* experiments as a next step towards a possible clinical exploration of the described approaches in the frame of de-intensification trials in HPV-positive HNSCC.

## Data Availability Statement

The original contributions presented in the study are included in the article/**Supplementary Material**. Further inquiries can be directed to the corresponding author.

## Author Contributions 

KH, TB, AO, SC, and FG conducted experiments under the supervision of TR. KH, TB, AO, SC, FG, and TR analyzed the data. MK, KR, CP, CB, NS and TR contributed conception and design of the study; AO, NS, KR, and TR wrote the manuscript. All authors contributed to the article and approved the submitted version.

## Funding

This work was supported by the German Cancer Aid (Deutsche Krebshilfe, grant 70113259; KR, TR) and the German Federal Ministry of Education and Research (BMBF, grant 02NUK032; MK, KR, TR).

## Conflict of Interest

The authors declare that the research was conducted in the absence of any commercial or financial relationships that could be construed as a potential conflict of interest.
