# Supplementary material for: Dual Inhibition of PARP and the Intra-S/G2 Cell Cycle Checkpoints Results in Highly Effective Radiosensitization of HPV-Positive HNSCC Cells
Source: Front Oncol. 2021 Jul 20;11:683688. doi: 10.3389/fonc.2021.683688 (PMC8329549; doi:10.3389/fonc.2021.683688)
Supplement: Supplementary file 1 [file DataSheet_1.pdf]

Supplementary Material

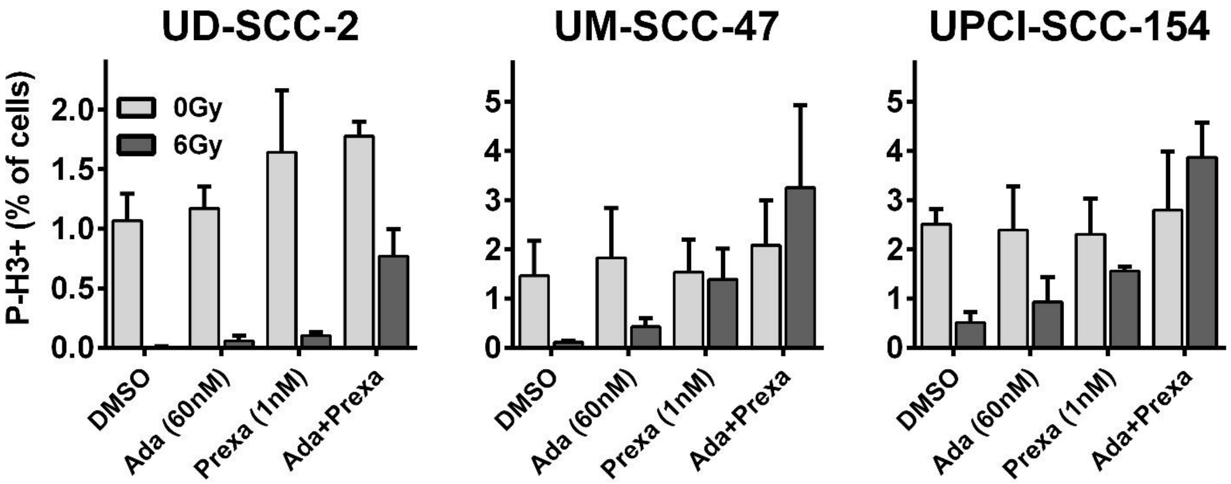

Supplementary Figure S1. Effectiveness of G2 arrest agrogation by low dose Wee1 and Chk1 or combined inhibition. Fraction of mitotic cells. Exponentially growing cells were treated for 2 hours with the inhibitors as indicated, before irradiation with 0 or 6 Gy. Eight h after irradiation cells were fixed and stained for the mitosis marker phospho-histone H3 (P-H3) to assess the number of mitotic cells. Enhanced mitotic fractions upon dual treatment and irradiation may be explained by aberrant mitotic divisions of cells entering mitosis with high levels of DNA damage.

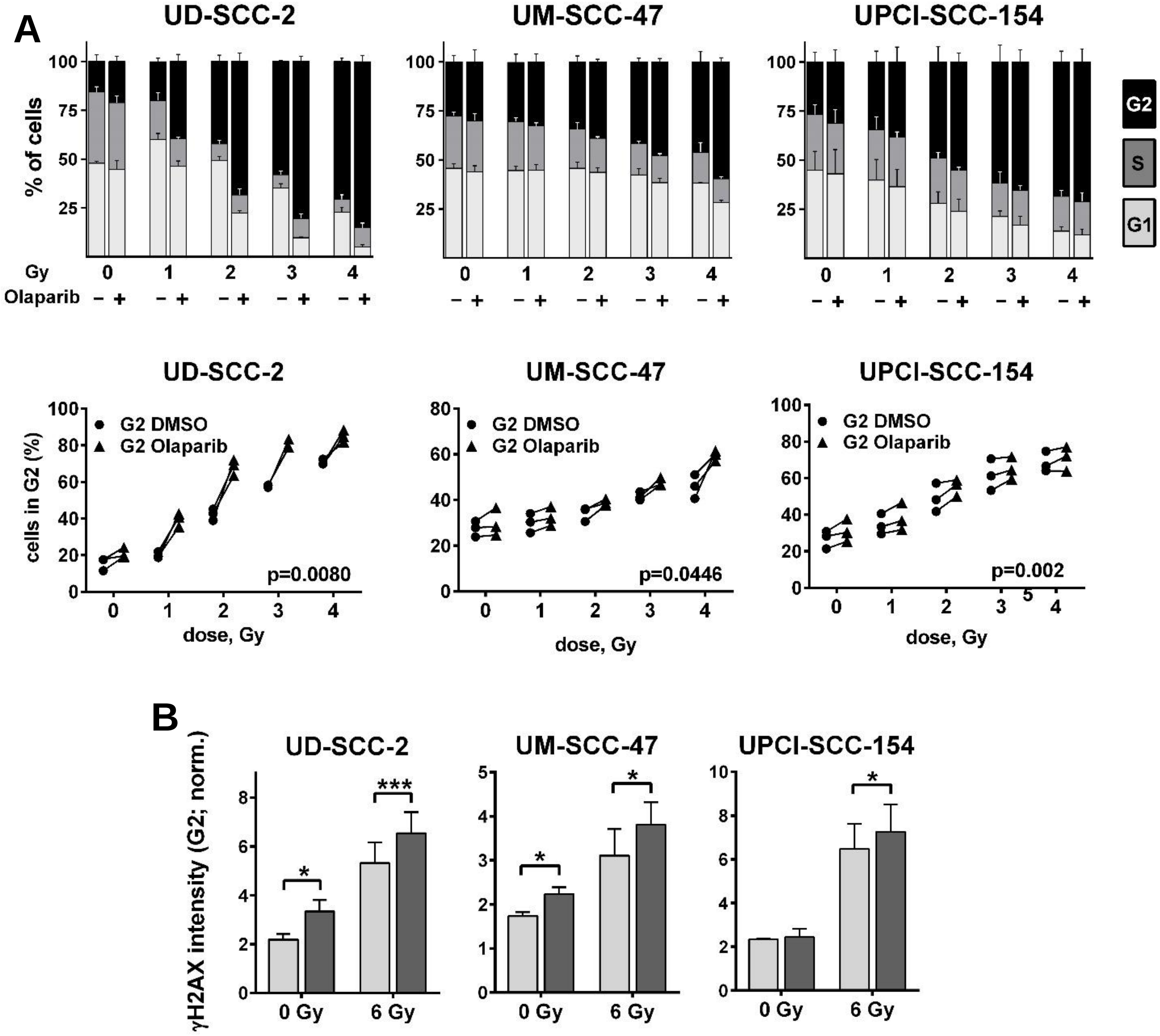

Supplementary Figure S2. Enhanced fractions of G2 phase cells and enhanced γH2AX levels in G2 arrested cells after PARP inhibition. (A) Top: Exponentially growing cells were treated and irradiated as indicated. Twenty-four h after irradiation the cells were fixed and the cell cycle distribution assessed by DAPI staining and flow cytometry. Bottom: While the increase of G2 phase cells upon olaparib treatment in UM-SCC-47 and UPCI-SCC-154 was subtle, the difference was seen in virtually every experiment and reached significance when including all doses (paired two-tailed Student's t-test). (B) Exponentially growing cells were treated with olaparib and after 2 h irradiated ± 6 Gy. Twenty-four h later, when the majority of irradiated cells is arrested in G2, the cells were fixed and DNA damage levels assessed by flow cytometric measurement of γH2AX levels plus DNA content (DAPI). Olaparib induced a significant increase in DNA damage levels in all strains in G2 arrested cells 24 h after 6 Gy irradiation and in 2/3 cell lines without irradiation (paired two-tailed Student's t-test). Depicted are the average values of the median γH2AX intensities of G2 phase cells, individual values were normalized to the γH2AX level of the non-irradiated G1 phase populations of the respective experiments.

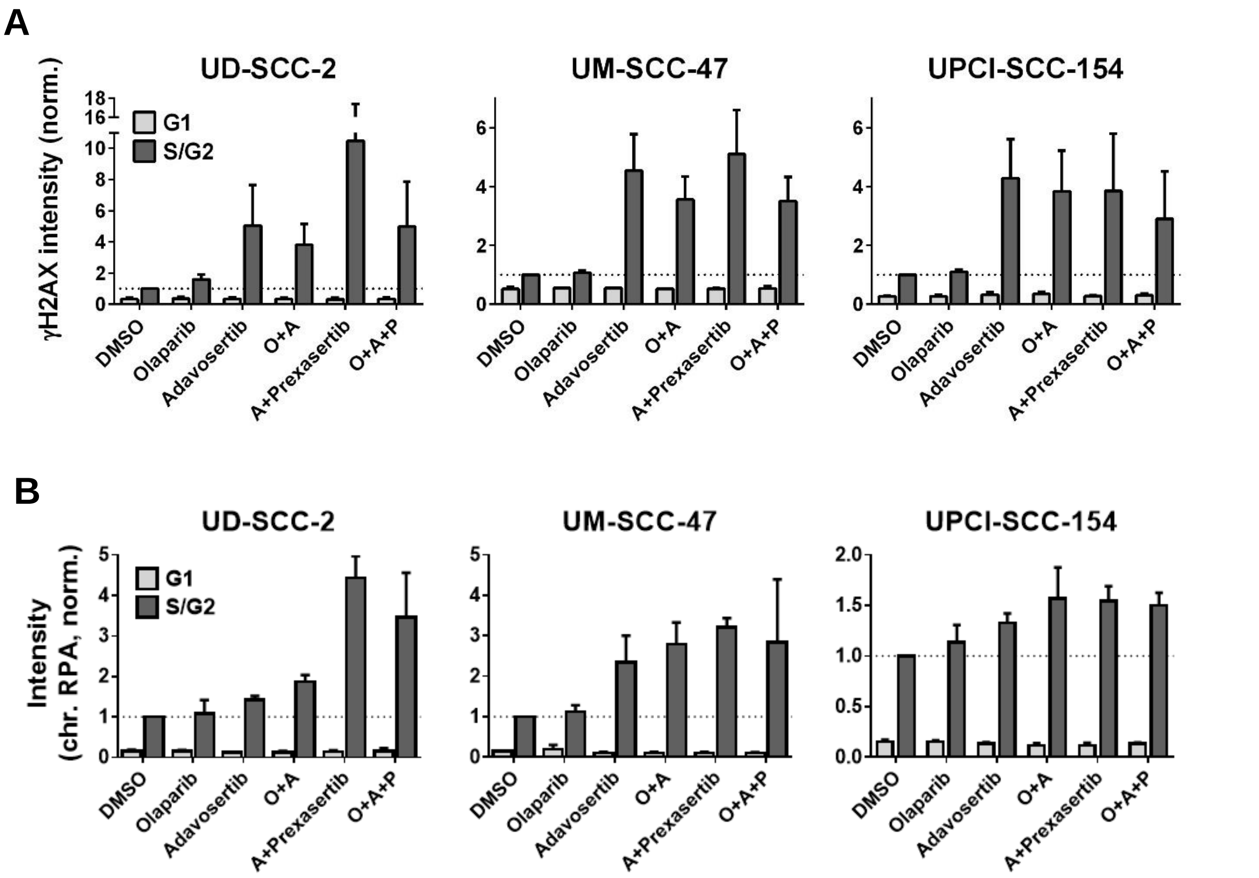

Supplementary Figure S3. Effect of PARP- and intra-S/G2 checkpoint inhibition on γH2AX and chromatin-bound RPA staining intensity. Data are based on the same experiments as for Figure 3. Graphs depict the staining intensities of G1 and S/G2-phase cells (green or blue + red populations in Figure 3A,C). (A) γH2AX. (B) Chromatin-bound RPA. Values were normalized to the solvent control (DMSO) value of the S/G2 population in each experiment.

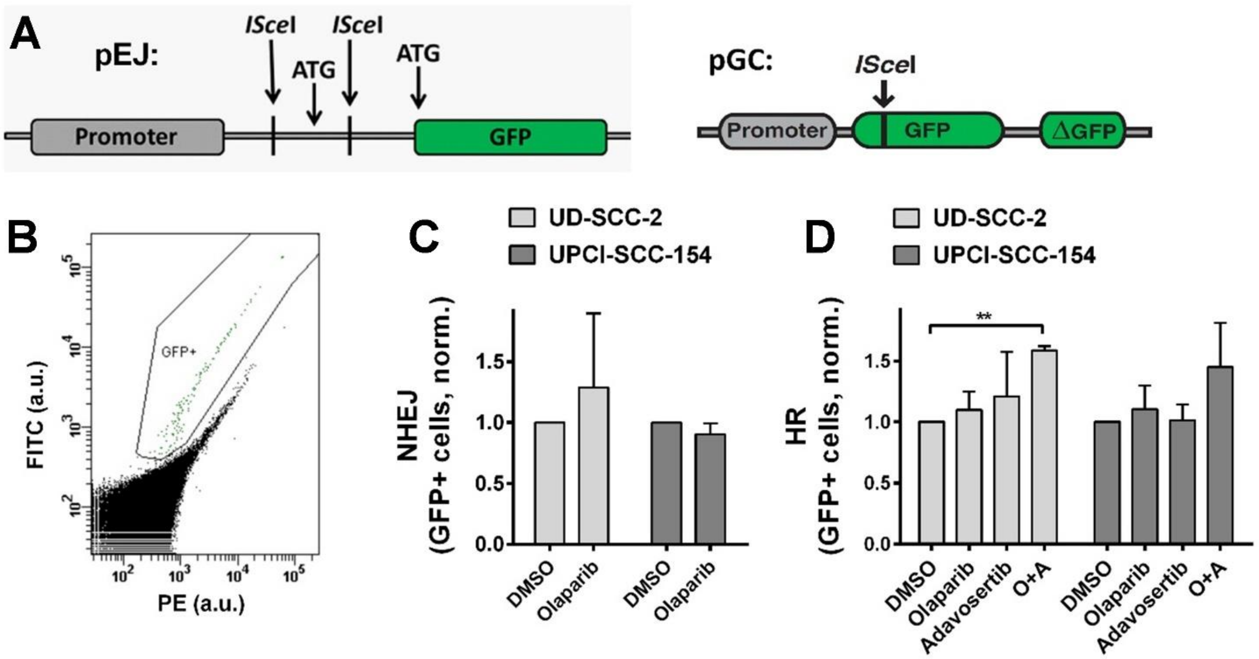

Supplementary Figure S4. Effect of PARP and Wee1 targeting on DSB repair pathways as assessed by GFP-based reporter gene assays. Reporter strains with stable integration of the respective repair constructs were transfected with an I-SceI expression vector. Six hours and once more 24 h after transfection the medium was exchanged and inhibitors added; 48h after transfection the fraction of GFP-positive cells was assessed by flow cytometry. **(A)** Scheme of the respective NHEJ and HR reporter constructs. **(B)** Example of flow cytometric assessment. **(C)** Effect of PARP inhibition on NHEJ efficacy and **(D)** of PARP and Wee1 inhibition on HR efficacy. Results were generally normalized to the respective transfection efficiencies and DMSO controls of the individual experiments.

Significant changes are indicated with ** indicating $p<0.01$, respectively (two-tailed Student's t-test).

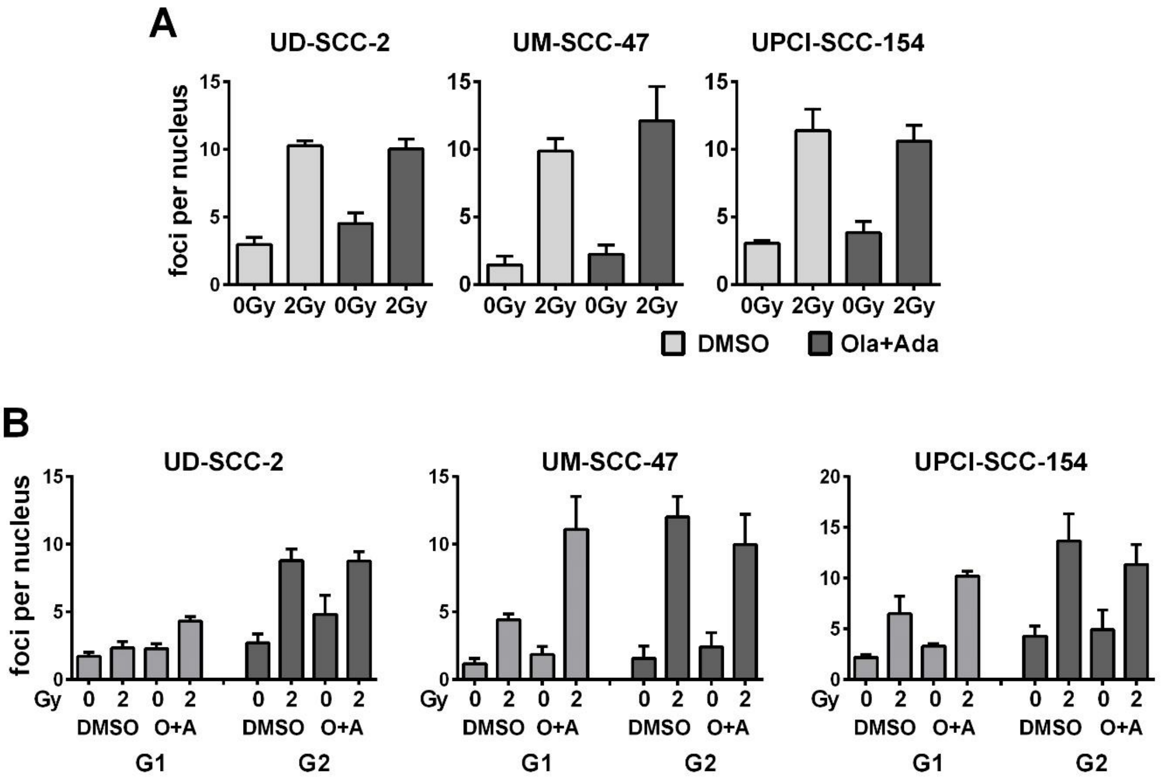

Supplementary Figure S5. Radiation-induced 53BP1 foci. Quantification of nuclear 53BP1 foci at 24 h after 0 & 2 Gy irradiation. Graphs are based on the same experiments as presented in Figure 4, for which the non-irradiated background levels of each experiment (0 Gy) had been subtracted. (A) Radiation-induced 53BP1 foci in all cell cycle phases. (B) Radiation-induced 53BP1 foci in G1 vs. S/G2 phase as assessed by geminin co-staining.

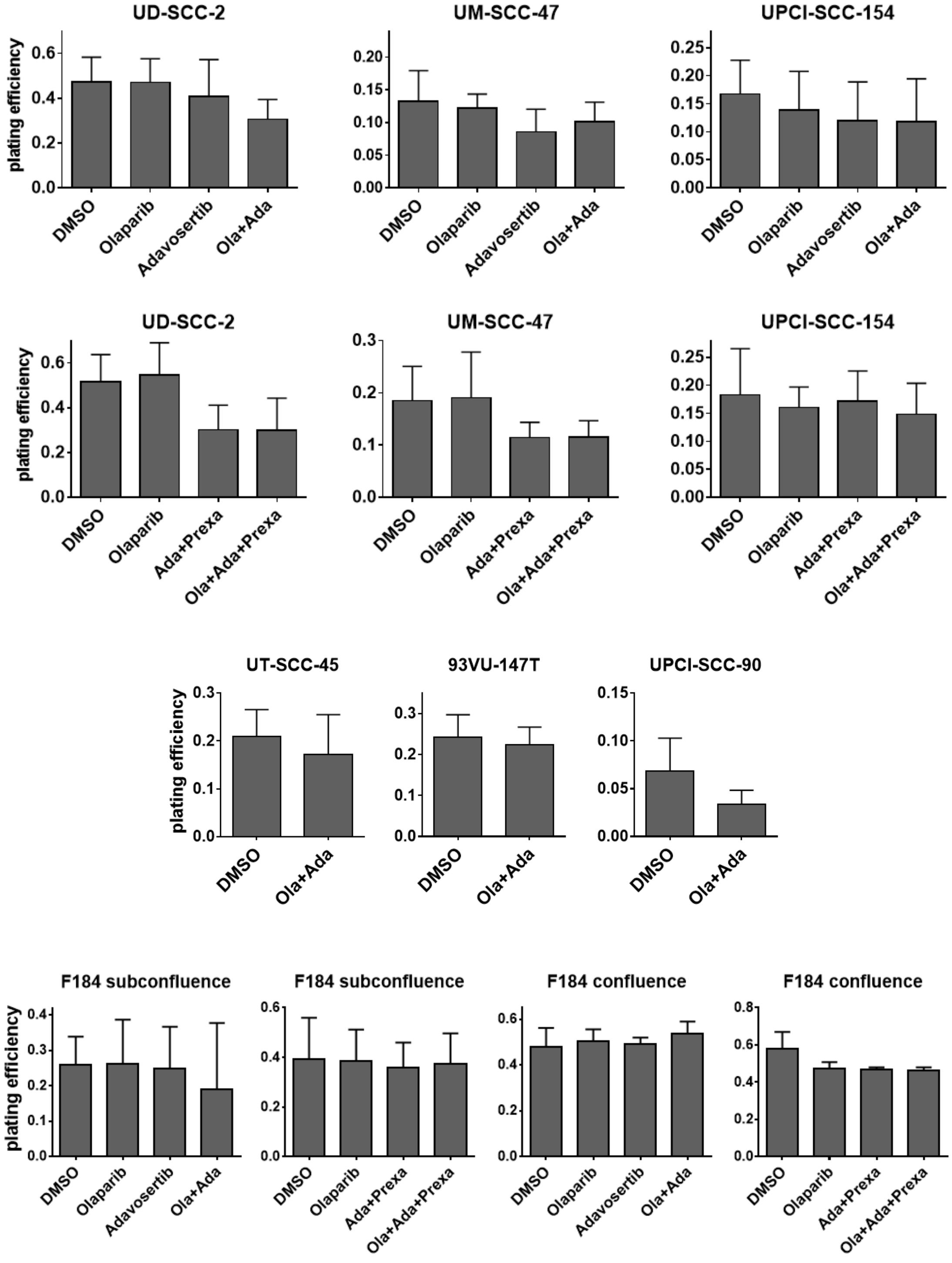

Supplementary Figure S6. Plating efficiencies of colony formation assays. Exponentially growing cells were treated with inhibitors as indicated and after 26 h seeded in defined, low numbers without inhibitors for colony formation. Graphs represent the non-normalized surviving fractions of the 0 Gy samples from the experiments shown in Figure 5.

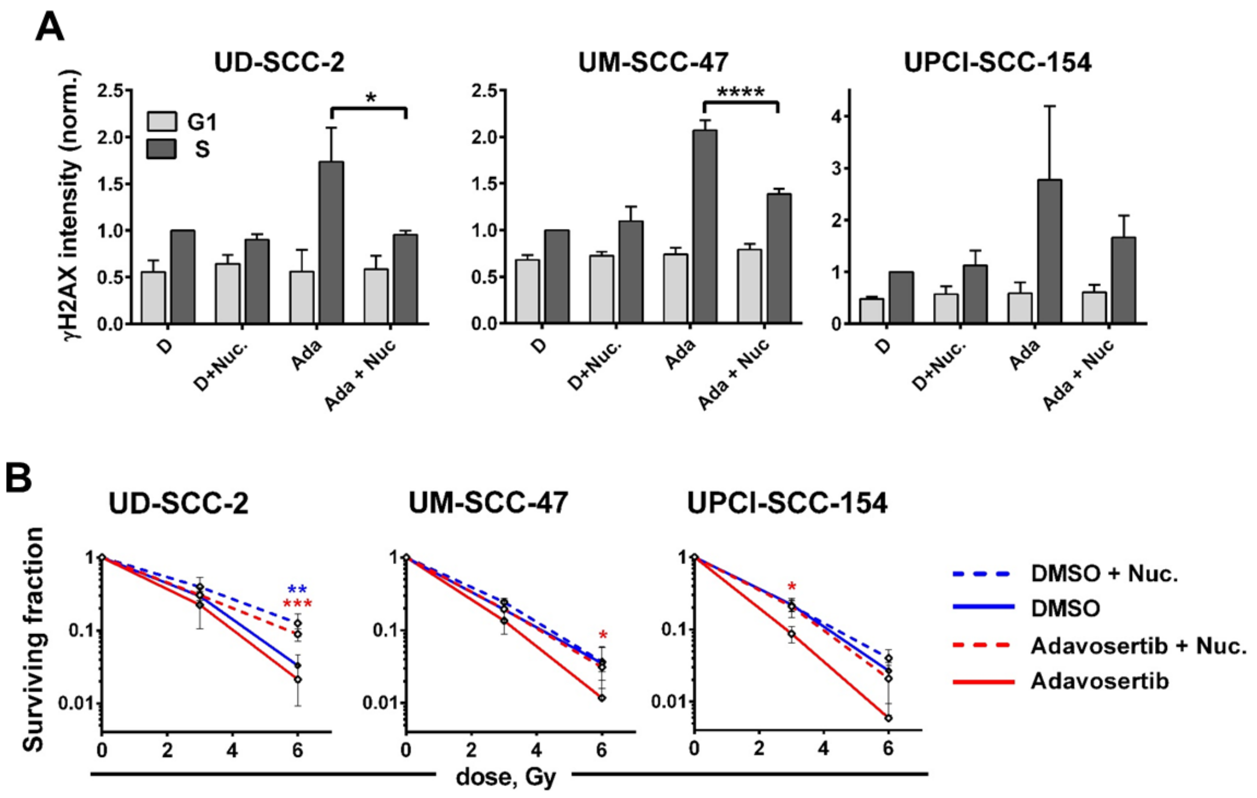

Supplementary Figure S7. Effect of nucleoside supplementation on radiosensitization through sole Wee1 inhibition. (A) Cells were treated with adavosertib ± nucleoside supplementation as indicated. After 4 h of treatment the cells were fixed and analyzed for γH2AX induction by flow cytometry. Bars depict the average median γH2AX staining intensity of cells in G1 and mid-S phase as assessed by DAPI co-staining. Values were normalized to the intensity of DMSO treated mid-S phase cells of the respective experiments Asterisks mark statistically significant differences upon nucleoside supplementation. **(B)** Cells were irradiated 2 h after addition of adavosertib ± nucleosides and after further 24 h seeded for colony formation without addition of inhibitor or nucleosides. Asterisks mark statistically significant differences in survival upon nucleoside supplementation, color indicates solvent controls or inhibitor treatment. Differences between DMSO and adavosertib treatment reached significance only in UPCI-SCC-154 cells (not indicated).

Significant changes are indicated with *, ** and *** indicating $p \leq 0.05$, $p \leq 0.01$ and $p \leq 0.001$, respectively (two-tailed Student`s t-test).

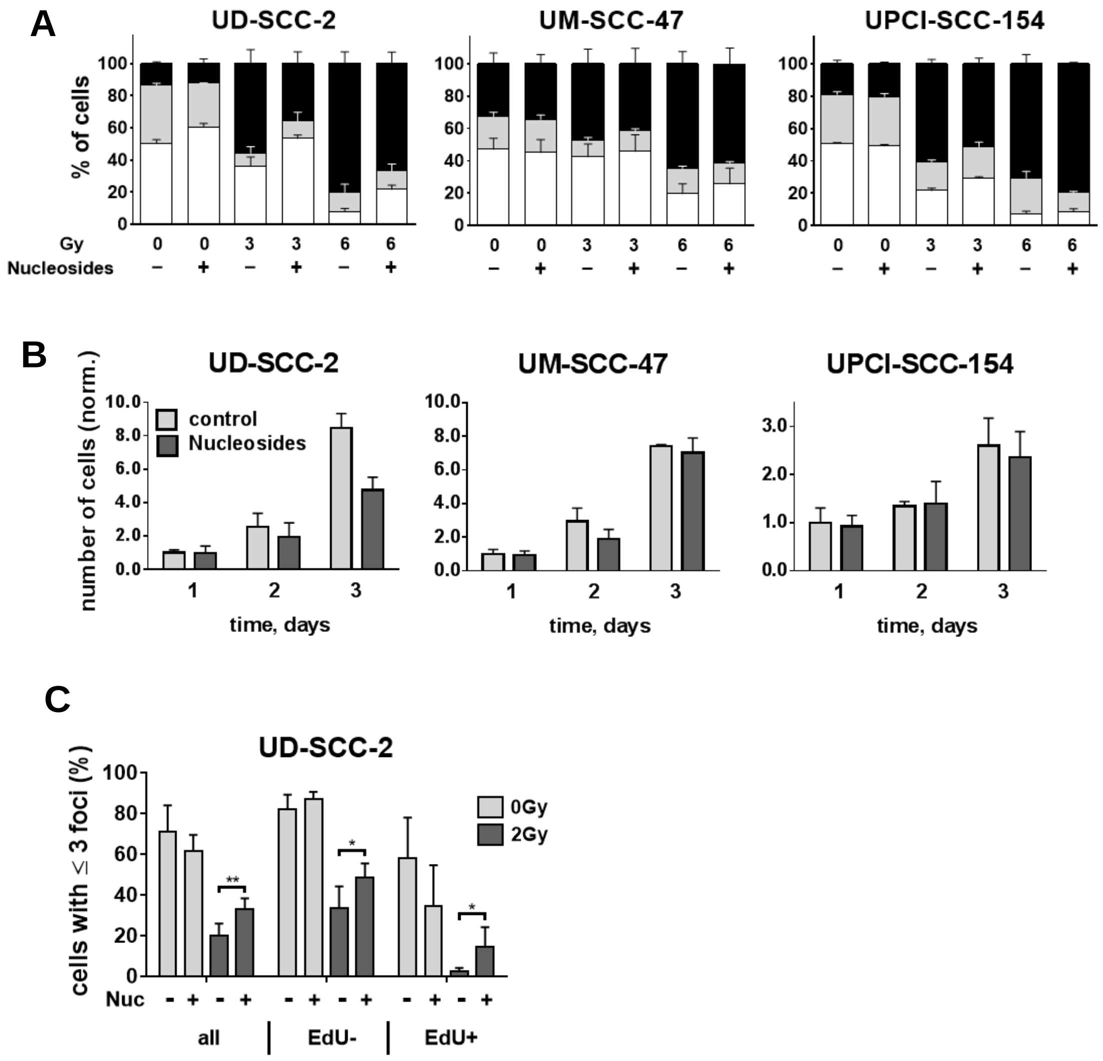

Supplementary Figure S8. Induction of radioresistance through nucleoside supplementation.
(A) Exponentially growing cells were treated with nucleosides and inhibitors as indicated and were irradiated after 2 h. Twenty-four hours later the cells were fixed and the cell cycle distribution assessed. (B) Exponentially growing cells were seeded and after 4 h treated with or without nucleosides and the resulting numbers of control treated cells were assessed at the indicated time points. Data are normalized to the resulting cell number after 24 h (day 1). (C) Exponentially growing cells were pulse labelled with EdU for 30 min, washed 4 times, supplemented with nucleosides and after further 30 min irradiated as indicated. Cells were finally fixed 24 h after irradiation and stained for 53BP1, DAPI and EdU. Cells showing few foci (≤3) are rare in the fraction of UD-SCC-2 cells irradiated in/immediately after S phase (EdU+). Nucleoside supplementation increases the proportion of cells with few foci in the fraction of cells irradiated in (EdU+) but also of those cells irradiated outside (EdU-) of S phase. Asterisks in (C) depict significant differences upon nucleoside supplementation with * and ** indicating $p<0.05$, $p<0.01$, respectively (two-tailed Student's t-test).

Cell line /treatment	DEF (25%)	CKEF (3Gy)
UD-SCC-2 / Ola+Ada	1.92	3.23
UM-SCC-47 / Ola+Ada	1.64	3.50
UPCI-SCC-154 / Ola+Ada	1.50	2.62
UT-SCC-45 / Ola+Ada	2.24	3.92
93VU-147T / Ola+Ada	1.85	3.01
UPCI-SCC-90 / Ola+Ada	1.51	1.5
F184 /Ola+Ada, subconf.	1.29	1.34
F184 /Ola+Ada, conf.	1.22	1.35
UD-SCC-2 / Ola+Ada+Prexa	2.15	4.35
UM-SCC-47 / Ola+Ada+Prexa	1.95	3.85
UPCI-SCC-154 / Ola+Ada+Prexa	2.54	9.85
F184 /Ola+Ada+Prexa, subconfl.	1.38	1.41
F184 /Ola+Ada+Prexa, confl.	1.06	0.89

Supplementary Table S1. Dose enhancement at 25% survival and cell kill enhancement at 3 Gy. The Dose Enhancement Factor (DEF) at 25% survival was directly assessed from the dose response curves depicted in Figure 5 using GraphPad Prism. The Cell Kill Enhancement Factor (CKEF) at 3 Gy was either calculated directly from the 3 Gy dose points of the dose response curves (see Figure 5) or, in case a 3 Gy dose point was lacking (UT-SCC-45, 93VU-147T and UPCI-SCC-90), was assessed by interpolation using GraphPad Prism.
